# Is Two Better Than One? Anticipation of a Favorable Hemodynamic Profile by Combining the Hypnotic Drugs for Induction of Anesthesia

**DOI:** 10.15171/jcvtr.2015.39

**Published:** 2015-11-29

**Authors:** Nader D. Nader

**Affiliations:** Department of Anesthesiology, University at Buffalo, Buffalo, NY, USA

Hypnosis is the main hallmark of the anesthesia for surgery and any other noxious procedure done in a hospital setting. The level of hypnosis is determined by the extent of noxious stimulation and the availability of supplementation with local/regional anesthesia. The level of anesthesia that prevents the patients from moving in response to the painful stimuli is universally achieved by general anesthesia.

During induction of general anesthesia the patients go through 4 stages from loss of consciousness to cessation of breathing. However, with introduction of rapidly acting intravenous hypnotic agents, transition of patients through these stages is hardly noticeable. Therefore the stage of excitement and delirium is seldom detected during induction of general anesthesia. This stage is almost exclusively seen in patients who are inadvertently overdosed during moderate sedation with propofol, nowadays.

With advances in safety of the anesthetic agents and evolved monitoring techniques, many more patients undergo invasive interventions who would have originally been denied of anesthesia because of their physiological limitation and severity of their illnesses. As we provide anesthetic care to patients with compromised cardiovascular reserve, a delicate balance should be maintained to preserve the myocardial contractility and systemic vascular resistance as much as possible. Most of the induction agents that are in clinical use, negatively affect cardiac inotropy and cause significant drops in the left ventricular preload and afterload.

Barbiturates are the oldest class of hypnotic agents that have been used for intravenous induction of anesthesia. They share the advantage of rapid onset of action and due to their shorter distribution half-life, they do not produce noticeable prolongation of recovery from anesthesia following a single dose administration. However, repeated doses of barbiturates or continuous intravenous administration of these drugs significantly prolong the recovery due to their longer elimination and context sensitive half-lives. Hyperalgesic effects and delayed recovery have limited the use of barbiturates in moderate sedation cases. Additionally, barbiturates posses a significant negative inotropy and venodilatory effects which causes remarkable drops in arterial blood pressures in patients with hypovolemia and preexisting systolic heart failure. Because of these pharmacologic characteristics, the use of barbiturates is not favored in cardiac anesthesia.

Ketamine differs from barbiturates in its ability to stimulate sympathetic activity and thereby increasing systemic vascular resistance and maintaining blood pressures during induction. It is important to note that in patients with prolonged untreated congestive heart failure where there is a depletion of the sympathetic tone, administration of ketamine may inadvertently cause myocardial suppression and lead to clinical hypotension during the induction of anesthesia. Additional lack of motivation in using ketamine in cardiac patients is due to the increases in myocardial oxygen consumption which is unmatched by limited supply in patients with ischemic heart diseases. Postoperative delirium and hallucination are also frequently reported after clinical use of ketamine that have further limited the use of this anesthetic induction agent.

Propofol is the most commonly used induction agent in clinical anesthesia practice among general population. Both induction and recovery are pleasant and welcomed by the patients. Due to shorter half-life, recovery from anesthesia even in patients who have received prolonged infusion of propofol is extremely brief. Propofol infusion is also commonly used for moderate sedation in patients undergoing invasive interventions outside of the operating room settings. The major disadvantage of propofol is its strong vasodilatory effects, which may cause significant drops of the left ventricular afterload and hypotension upon induction of anesthesia.

Etomidate is most commonly reserved for patients with limited cardiac function because it preserves myocardial contractility and systemic vascular resistance and therefore provide a favorable hemodynamic profile. In clinical cardiac anesthesia practice, etomidate is probably the most commonly used single induction agent. Inhibition of the hypothalamic pituitary adrenal axis by etomidate and related role in inhibiting the body homeostasis in adrenal stress response is probably the biggest shortcoming of this drug in cardiac patients.

In this issue of *Journal of Cardiovascular and Thoracic Research*, Yagan et al have randomized 90 relatively healthy patients to receive etomidate (0.3 mg/kg), propofol (2.5 mg/kg) or a combination of etomidate and propofol (propofol dose of 1.25 mg/kg + etomidate dose of 0.15 mg/kg) for the induction of anesthesia.^1^ Mean arterial blood pressure (MAP), heart rate and rate pressure product (HR*MAP) were measured at 10 different time points prior an after tracheal intubation. MAPs were better maintained in the combination formula compared to propofol at the same time the combination formula prevented the surges of MAP during intubation compared to etomidate. One may conclude that combining propofol with etomidate while it prevented inadvertent hypotension during induction it blocked the hemodynamic response to the tracheal stimulation.

Clinical anesthesiologists have tried to achieve a better hemodynamic profile by combining various induction agents to synergistically produce hypnosis and avoid their individual side effects. Propofol has been generally used as a common ingredient of these combination therapies. This article suffers from the fact that only healthy (ASA I) patients and those with mild systemic diseases (ASA II) were included in randomization. Another recent study has enrolled 100 cardiac patients with reduced left ventricular ejection fraction. The hemodynamic profile at the time of induction with a combination of propofol with ketamine was similar to those receiving etomidate and benzodiazepines.^2^ Despite these promising finding, one should remember that the pharmaceutical concerns of mixing two drugs with different physical properties (emulsion forms) need to be extensively studied and addressed before the use of combination induction formulas could be recommended.

## Ethical issues

Not applicable.

## Competing interests

Author declares no conflict of interest in this study.
